# Monoamine Oxidase (MAO) Is Expressed at the Level of Mitral Valve with Severe Regurgitation in Hypertrophic Obstructive Cardiomyopathy: A Case Report

**DOI:** 10.3390/medicina58121844

**Published:** 2022-12-15

**Authors:** Ana Lascu, Raluca Șoșdean, Loredana Nicoleta Ionică, Alexandru S. Pescariu, Lucian Petrescu, Adina Ionac, Constantin T. Luca, Adrian Sturza, Horea B. Feier

**Affiliations:** 1 Department III—Pathophysiology, “Victor Babeș” University of Medicine and Pharmacy of Timișoara, E. Murgu Sq. No. 2, 300041 Timișoara, Romania; 2 Center for Translational Research and Systems Medicine, “Victor Babeș” University of Medicine and Pharmacy of Timișoara, E. Murgu Sq. No. 2, 300041 Timișoara, Romania; 3 Department VI—Cardiology, “Victor Babeș” University of Medicine and Pharmacy of Timișoara, E. Murgu Sq. No. 2, 300041 Timișoara, Romania; 4 Research Center of the Institute for Cardiovascular Diseases, “Victor Babeș” University of Medicine and Pharmacy from Timișoara, G. Adam St. No. 13A, 300310 Timișoara, Romania; 5 Doctoral School Medicine-Pharmacy, “Victor Babeș” University of Medicine and Pharmacy Timișoara, E. Murgu Sq. No. 2, 300041 Timișoara, Romania; 6 Department VI—Cardiovascular Surgery, “Victor Babeș” University of Medicine and Pharmacy of Timișoara, E. Murgu Sq. No. 2, 300041 Timișoara, Romania

**Keywords:** hypertrophic obstructive cardiomyopathy, mitral valve regurgitation, monoamine oxidase, oxidative stress, MAO inhibitors

## Abstract

Hypertrophic obstructive cardiomyopathy (HOCM) is one of the most common hereditary heart diseases. The severely hypertrophied interventricular septum combined with the systolic anterior movement (SAM) of the mitral valve (MV) frequently cause a significant pressure gradient in the left ventricular outflow tract associated with varying degrees of mitral regurgitation (MR). We present the case of a 64-year-old female patient who was diagnosed with HOCM two years ago and was admitted to the Institute of Cardiovascular Disease with exertion dyspnea and fatigue. Transthoracic echocardiography revealed concentric, asymmetrical left ventricular hypertrophy, an elongated anterior mitral leaflet (AML) and a significant SAM causing severe regurgitation, with indication for valvular replacement Monoamine oxidase (MAO), a mitochondrial enzyme, with 2 isoforms, MAO-A and B, has emerged as an important source of reactive oxygen species (ROS) in the cardiovascular system, but literature data on its expression in valvular tissue is scarce. Therefore, we assessed MAO-A and B gene (qPCR) and protein (immune fluorescence) expression as well as ROS production (spectrophotometry and confocal microscopy) and in the explanted MV harvested during replacement surgery. MAO expression and ROS production (assessed by both methods) were further augmented following ex vivo incubation with angiotensin II, an effect that was reversed in the presence of either MAO-A (clorgyline) or B (selegiline) inhibitor, respectively. In conclusion, MAO isoforms are expressed at the level of severely impaired mitral valve in the setting of HOCM and can be induced in conditions that mimic the activation of renin-angiotensin-aldosterone system. The observation that the enzyme can be modulated by MAO inhibitors warrants further investigation in a patient cohort.

## 1. Introduction

Hypertrophic obstructive cardiomyopathy is one of the most frequent genetic diseases of the heart muscle [1]. The severely hypertrophied interventricular septum along with the systolic anterior movement (SAM) of the anterior mitral leaflet (AML) often induce a significant pressure gradient in the left ventricular outflow tract (LVOT) along with variable degrees of mitral regurgitation. The shear stress on the mitral valve apparatus in this process may activate molecular mechanisms that induce early degeneration of the valvular tissue, additionally altering the mitral valve function. Patients with a persistent significant LVOT pressure gradient and a severe mitral regurgitation due to additional mechanisms besides SAM of AML have a more intense symptomatology. They need heart failure therapy including diuretics, which may enhance the LVOT pressure gradient. Additionally, these patients need surgical intervention for mitral valve replacement and possible septal reduction therapy by myectomy [2].

Oxidative stress has been systematically reported as a central pathomechanism in cardiac diseases [3] and there is an increased interest for the characterization of reactive oxygen species (ROS)-induced metabolic pathways in cardiomyocytes in order to identify additional treatment strategies [4]. However, there are few literature data about reactive ROS generation in the abnormal valvular tissue. The group of Donald Heistad firstly reported that oxidative stress was increased in calcified regions of stenotic aortic valves removed during surgical valvular replacement due to the dysregulation of the antioxidant defense [5]. We have previously reported an increase in local ROS at the level of the diseased mitral valve harvested during surgical replacement from a young patient with primary severe mitral regurgitation; interestingly, the magnitude of oxidative was mitigated after treating the sample with an angiotensin II type 1 (AT1) receptor antagonist [6].

The past two decades witnessed an increasing interest for the contribution of mitochondria-derived ROS since these organelles are particularly abundant in the heart [7,8]. Monoamine oxidase (MAO) is a mitochondrial enzyme with two isoforms, MAO-A and MAO-B, which catalyze the oxidative deamination of biogenic monoamines and neurotransmitters with the constant generation of the corresponding aldehydes, ammonia, and H_2_O_2_. MAO has recently emerged as an important contributor to the cardiovascular oxidative stress in both animal models and humans [9,10,11,12]. MAO-A is the predominant isoform in the cardiovascular systems of both humans and rats, and its expression has been reported to increase with the age [13], e.g., there is 6-fold MAO-A in the aged rat heart [14]. Peña-Silva et al. firstly identified MAO-A as being responsible for valvular oxidative stress in valves harvested from human explanted hearts that were not used for transplantation were exposed to serotonin and dopamine [15].

We report here the case of a female patient who was diagnosed with hypertrophic obstructive cardiomyopathy and severe mitral regurgitation with indication for valve surgery. We found the expression of both MAO isoforms at the level of explanted mitral valve with the predominance of MAO-A, which contributes to the local oxidative stress and can be modulated by MAO inhibitors.

## 2. Case Presentation

A 64-year-old female patient, with grade II essential arterial hypertension and hypercholesterolemia, diagnosed with hypertrophic obstructive cardiomyopathy ~2 years ago, under maximal tolerated beta-blocker therapy and diuretic therapy (loop diuretic and mineralocorticoid receptor blocker), presented to the clinic for fatigue and exertional dyspnoea.

The resting electrocardiogram (ECG) at admission revealed sinus rhythm with a heart rate of 64 beats/minute, a Sokolov-Lyon index of 36 mm, with repolarization abnormalities in the lateral leads, consistent with left ventricular (LV) hypertrophy.

**Figure 1:**
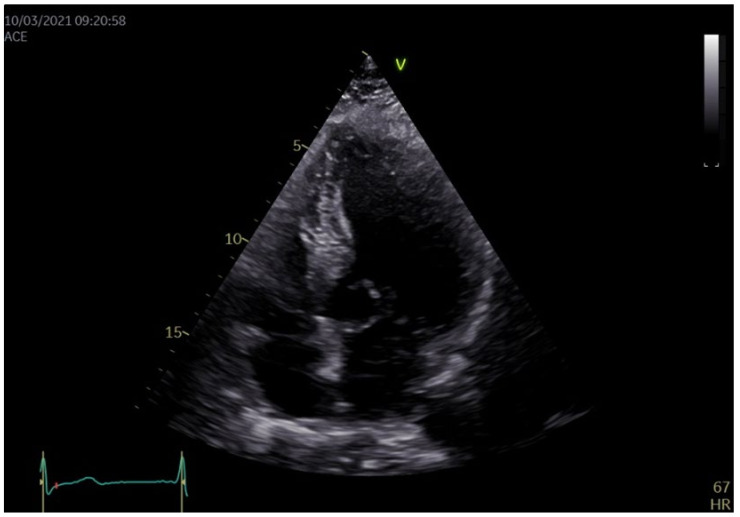
Transthoracic bidimensional echocardiography, 4 chamber view: a mitral valve with fibrosed leaflets, an elongated AML and SAM of both AML and PML, creating an anteriorly deviated “funnel”.

**Figure 2:**
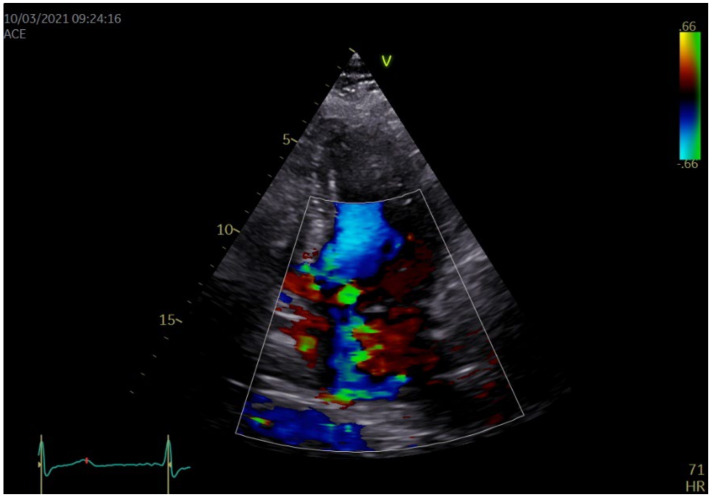
Transthoracic bidimensional echocardiography, four chamber view with color Doppler on the mitral valve: severe mitral valve regurgitation, with a central slightly posterolateral deviated jet. The regurgitant jet enters the pulmonary veins.

Echocardiography revealed a concentric, asymmetrical left ventricular (LV) hypertrophy, with normal dimensions of the LV cavity and a normal ejection fraction (LVEF). Additionally, the right ventricular free wall had an increased thickness, consistent with an associated RV hypertrophy, but with a preserved systolic function. The mitral valve (Figure 1) had an elongated anterior leaflet (AML), with a significant systolic anterior movement (SAM) of more than 30% of the systolic time. The SAM could also be seen on the short posterior mitral leaflet (PML), forming an anteriorly oriented “funnel” with the AML. Fibrosis was observed on both leaflets with calcification of the posterior mitral valve ring, inducing a severe regurgitation with a central, slightly posterolateral oriented jet (Figure 2).

The pressure gradient in the left ventricular outflow tract was measured to be 100 mmHg, without any provoking method. The most important echocardiographic parameters are presented in Table 1.

Given the association of a significant interventricular hypertrophy, a significant left ventricular outflow tract (LVOT) gradient despite maximal tolerated medical treatment and a severe mitral regurgitation induced by SAM and early degenerative lesions, valvular replacement therapy was decided as the most appropriate management. The intervention was performed without intraoperative and/or postoperative complications. The intraoperative view of the mitral valve confirmed the echocardiographic description, revealing fibrotic and thickened leaflets, with an elongated anterior leaflet and a calcified posterior ring. The mitral valve was replaced with a mechanical prosthesis (Sorin Carbomedics Top Hat number 27 mm), due to the high risk of LVOT obstruction by the biological prosthesis stents. Mitral valve replacement with a mechanical prosthesis was considered to the best therapeutic approach for the management of both severe mitral regurgitation and LVOT obstruction.

The explanted diseased valvular tissue was sent to the Center for Translational Research and Systems Medicine and used for the assessment of: (i) monoamine oxidase (MAO) gene (qRT-PCR, DNA gel electrophoresis) and protein (immune-fluorescence) expressions and (ii) ROS production by means of dihydroethidium (DHE) staining (confocal microscopy) and Ferrous Oxidation-Xylenol Orange (FOX) assay (spectrophotometry), respectively. The valvular tissue was incubated (EBM + 1% BSA, 37 °C) with/without angiotensin II (AII, 100 nM, 12 h) in the presence vs. absence of MAO-A (clorgyline, 10 µM) and B (selegiline, 10 µM) inhibitor.

**Figure 3:**
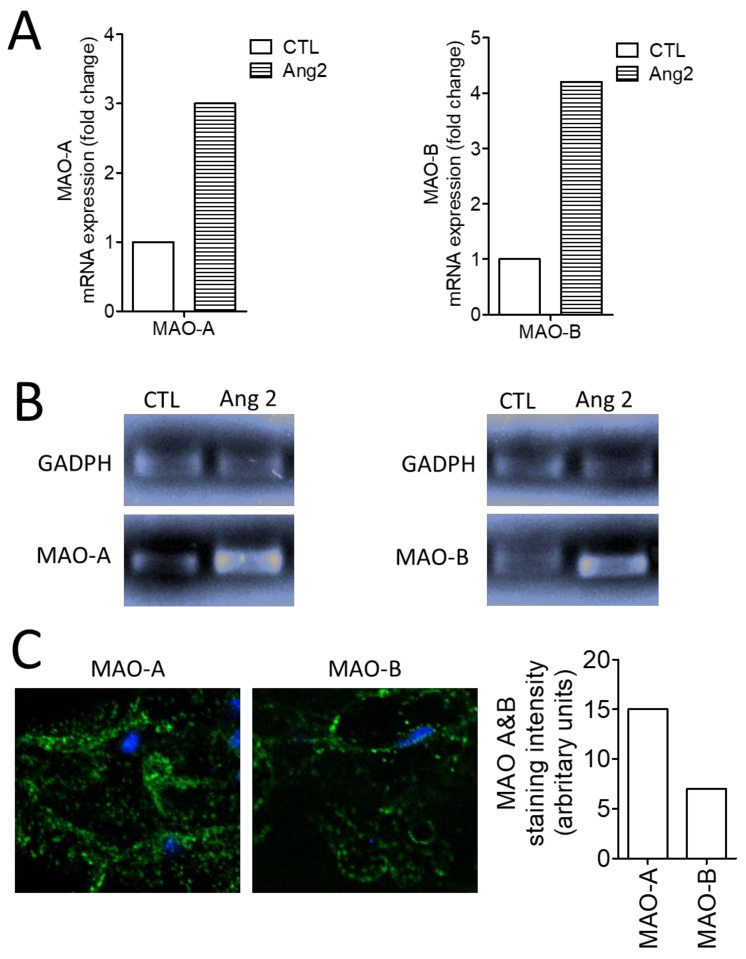
Expression of MAO-A and MAO-B in valvular tissue. (**A**) qRT-PCR for MAO-A and MAO-B mRNA expression (relative gene: GADPH), (**B**) DNA gel electrophoresis, (**C**) Immune-fluorescence for MAO-A and MAO-B protein expression. (CTL, control; AII, Angiotensin II).

Gene expression of both MAO isoforms was increased after AII stimulation as revealed by the mRNA level (assessed by qRT-PCR) and DNA gel electrophoresis (Figure 3A,B) and also the protein level (evaluated by immune fluorescence) (Figure 3C). Of note, a predominance of the MAO-A protein (vs. MAO-B) in the diseased valvular sample was evident in the immune fluorescence.

**Figure 4:**
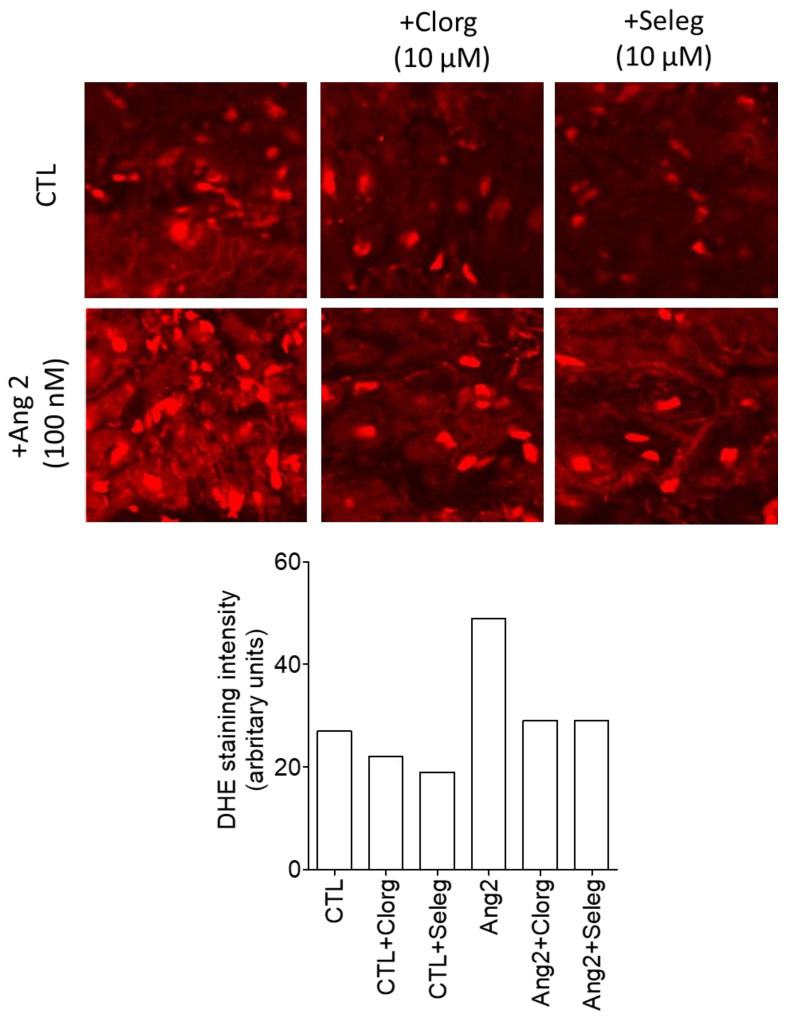
Assessment of superoxide (bright red) in the explanted mitral valve by confocal microscopy (DHE staining) in the presence of AII, clorgyline, and selegiline.

As for the ROS quantification, DHE staining—used to assess the superoxide anion (Figure 4) and FOX assay—which measures hydrogen peroxide (Table 2) revealed the presence of both species in the explanted valvular tissue. The amount was further increased after incubation with AII and decreased after the addition of either the MAO-A (clorgyline) or MAO-B (selegiline) inhibitor.

The postoperative evolution was favorable with a normal function of the mitral prosthesis and a LVOT pressure gradient of 15 mmHg. The improved clinical condition and investigation results were maintained at 3- and 6 months follow-up, respectively.

## 3. Discussion

We report here the case of a 64-year-old patient with HOCM and important interventricular septum (IVS) hypertrophy combined with the systolic anterior movement of the anterior mitral leaflet and severe mitral regurgitation that required valvular replacement therapy. The exact mechanism for SAM of the mitral valve is still debated. The SAM is more obvious for the anterior leaflet and less obvious for the shorter posterior mitral leaflet, which cannot travel that far, thus resulting in coaptation defect and a posterolateral regurgitant jet [16]. The important IVS hypertrophy and its strong contractility in HOCM induces LVOT narrowing, especially during the systole. This aspect will induce a high velocity systolic flow through the LVOT, with a high differential pressure between left atrium and LVOT that will deviate dominantly the anterior mitral leaflet towards the IVS (the Venturi effect), thus enhancing the obstruction and the pressure gradient in the LVOT [17]. On the other hand, the elongated AML or PML and the modified position of the papillary muscles, which are more apical and/or anterior and inward, will produce abnormal flow and pressure on the chordae tendineae, altering their tension, and anteriorly deviating one or both leaflets during the systole [18]. The degree of SAM appears to be related more to the abnormalities of the leaflets and less to the degree of IVS hypertrophy. An elongation of the mid-anterior part would cause an associated prolapse whereas a dominant elongation of the distal anterior part would cause an associated flap [19], as also seen in our patient. The mostly central jet of the regurgitation is suggestive for an additional mechanism, besides SAM of the mitral valve, which induces a typical posterolateral regurgitant jet, although in certain cases it may also be central and/or anteriorly oriented, depending on the AML-PML “funnel” orientation. However, the impaired flows and pressures will increase the mitral valve shear stress, especially in cases with significant SAM and significant secondary mitral valve regurgitation. Both SAM of the AML and age-dependent valvular degeneration induced a severe incompetence of the valvular coaptation. In these situations, if echocardiography shows that the mitral valve dominantly contributes to the LVOT obstruction (elongated AML, significantly modified apparatus, severe SAM of over 30% of the systolic time), valve replacement with a mechanical prosthesis (if no contraindications to permanent anticoagulation are detected) may be performed alone, without myectomy, with good results [20], as was in this case.

Hypertrophic cardiomyopathy (HCM) has a complex pathophysiology in which, besides inefficient sarcomere contraction, several additional disease hits contribute to its severity, among which metabolic changes leading to mitochondrial dysfunction and oxidative stress have been involved [21]. The current understanding of the disease postulates that the primary impairment of sarcomeric proteins elicits energy depletion that disturbs cellular metabolism and increases oxidative stress, one of the major sources of the latter being the dysfunctional mitochondrial electron transport chain, as recently described in a comprehensive review. These authors summarized the studies in the field that reported a significant increase in the oxidative stress markers, such as damage to DNA, proteins, and lipids, in both heart and serum, some of them being correlated with echographic parameters (left ventricular dilation, ejection fraction) [22].

A number of studies evaluated the magnitude of the *systemic* oxidative stress in patients with HCM. Among these, in a pioneering study Dimitrow et al. reported the occurrence of increased oxidative stress in patients with HCM evidenced by increased serum levels of 8-isoprostaglandin F(2alpha), which was the highest in the subgroup of HCM patients with LVOT obstruction [23]. More recently, Szygula-Jurkiewicz et al. evaluated the total oxidative-antioxidant balance in patients with HCM and showed that patients with HCM had prooxidative changes with predictive value as compared to controls [24].

Research performed in the past decade has mostly addressed the role of *cardiac* oxidative stress in both animals and humans with HCM. As such, Christiansen at al. reported both the impairment of oxidative phosphorylation and increased release of mitochondrial ROS in heart samples from cats with spontaneously occurring HCM and preserved LV systolic function [25]. A very recent comprehensive study reported that HCM hearts showed decreased high energy phosphates metabolites (ATP, ADP, and phosphocreatine), an increased fraction of severely damaged mitochondria with reduced cristae density, citrate synthase and mitochondrial oxidative respiration and impaired redox homeostasis [26]. Nowadays, it is known that the mitochondrial dysfunction in the setting of pathological hypertrophy is more complex with significant changes occurring not only in mitochondrial energetics and redox balance but also in the expression of nuclear and mitochondrially encoded transcripts and mitochondrial proteome composition [27].

The electron leakage associated with the decreased activity of the electron transport system complexes at the inner mitochondrial membrane is classically considered the main source of mitochondrial ROS in the heart. In the past two decades, MAO with 2 isoforms, A and B, at the outer mitochondrial membrane, which catalyze the oxidative deamination of monoamines, has emerged as an important contributor to oxidative stress via the constant generation of H_2_O_2_ in an increasing number of cardiac pathologies [28]. Both MAO isoforms also regulate their substrate concentration in the heart being relevant the one of serotonin, norepinephrine and dopamine, which together with the other degradation products induce inflammation, cellular hypertrophy, and production of extracellular matrix with fibrosis [5,9,13,29]. Angiotensin II (AII) has also well-described mitogenic and profibrotic actions in long run, resulting in cell growth and senescence, pathological heart hypertrophy, and cardiovascular remodeling. AII-induced ROS production has been more recently described and is partially understood [30].

In the present study, we assessed the presence of MAO in the diseased mitral valve. Both MAO isoforms were expressed at gene level in the explanted tissue, but the MAO-A isoform protein detected in immune fluorescence was more abundant as compared to MAO-B isoform. The local generation of both types of ROS, superoxide and hydrogen peroxide, was augmented by ex vivo incubation with AII, suggesting that the local activation of the RAAS in the heart also could contribute to the valvular oxidative stress.

Importantly, the valvular oxidative stress was mitigated by incubation with either the MAO-A inhibitor, clorgyline, or the MAO-B inhibitor, selegyline, an observation that indirectly suggests that AII might potentiate the MAO activity. This finding is in line with the pioneering work of Peña-Silva et al. regarding the contribution of MAO to the oxidative stress in human pulmonary and tricuspid valves. These authors investigated the contribution of serotonin to the valvular oxidative stress in explanted human valves incubated with tranylcypromine (an MAO-A/B inhibitor) and clorgyline (MAO-A inhibitor) and hypothesized that enzyme inhibition will prolong and augment the effects of serotonin (MAO substrate which will not be metabolized). To their surprise, MAO inhibition greatly reduced the valvular oxidative stress. At variance from their study where they added MAO substrates (serotonin, dopamine), we did not add any MAO substrates, which indicates that human pathological valvular tissue contains endogenous MAO substrates in sufficient amounts to allow the enzyme activity.

We have to acknowledge as study limitations that we did not assess the contribution to the valvular oxidative stress of other important ROS sources such as NADPH oxidase. Of note, in the abovementioned study, MAO inhibitors (tranylcypromine and clorgyline) did not affect the NADPH-related oxidative stress in the homogenates of heart valves.

Several lines of evidence unequivocally showed that mitochondrial oxidative stress contributes to the pathogenesis and progression of HCM. Characterization of the function of MAO in the valvular system along with the elucidation of the pathophysiological role of MAO-A in the setting of valvular defects/cardiomyopathies, represent one of the major challenges of the future. Interventions aimed at improving both redox balance and mitochondrial function are currently investigated in cardiovascular pathologies and may also be promising therapeutic approaches in hypertrophic cardiomyopathy. There is an armamentarium of MAO inhibitors, which are extensively used in the clinical practice and may become good candidates for drug repurposing in cardiovascular pathologies, including cardiomyopathies.

## 4. Conclusions

We have demonstrated here that MAO isoforms are expressed in explanted pathological mitral valve from a patient with hypertrophic obstructive cardiomyopathy and contribute to local oxidative stress; ROS production was further augmented by AII and mitigated by MAO inhibitors. Whether monoamine oxidase-related oxidative stress contribute to the pathogenesis of the disease in patients with hypertrophic obstructive cardiomyopathy and/or its complications, such as valvular derangement, warrants further investigation in a cohort of patients.

## Figures and Tables

**Table 1:** Presentation of the transthoracic echocardiographic parameters.

Parameter	Value/Structure and Function Alteration
**Chambers quantification**	
Interventricular septum (IVS), mm	21
Left ventricular posterior wall (LVPW), mm	15
Left ventricular end-diastolic diameter, mm	44
Left ventricular end-diastolic volume, mL	90
Left ventricular ejection fraction (LVEF), %	60
S’ mitral annulus IVS/lateral wall, m/s	0.08/0.10
LV global longitudinal strain, %	−19.3 altered in the basal segments
LV twist, degrees	8
Left ventricular outflow tract maximal pressure gradient, mmHg	100
Left atrium (LA) diameter, mm	49
Right ventricular free wall, mm	7
Right ventricular diameter, mm	27
Tricuspid annular plane systolic excursion (TAPSE), mm	25
S’ tricuspid annulus, m/s	0.16
Right ventricular fractional area change (RV FAC), %	40
**Valves quantification**	
Mitral valve	Severe regurgitation
Aortic valve	Degenerated
Tricuspid valve	Moderate regurgitation
Pulmonary systolic arterial pressure, mmHg	40
Pulmonary valve	Minor regurgitation

mL, milliliter; mm, millimeter.

**Table 2:** Assessment of H_2_O_2_ in the explanted mitral valve by spectrophotometry (FOX assay).

Tissue	H_2_O_2_ Amount
	(nM H_2_O_2_/mg/Tissue/h)
CTL	7.13
CTL + Seleg	6.2
CTL + AII	12.25
CTL + AII + Clorg	8.63
CTL + AII + Seleg	8.75

CTL, control; Seleg, Selegiline; AII, Angiotensin II; Clorg, Clorgyline.

## Data Availability

Data is contained within the article.
